# An Adaptive Transmitting Scheme for Interrupted Sampling Repeater Jamming Suppression

**DOI:** 10.3390/s17112480

**Published:** 2017-10-29

**Authors:** Chao Zhou, Feifeng Liu, Quanhua Liu

**Affiliations:** 1 Radar Research Laboratory, School of Information and Electronics, Beijing Institute of Technology, Beijing 100081, China; ericzc1987@163.com (C.Z.); liuquanhua@bit.edu.cn (Q.L.); 2 Key Laboratory of Electronic and Information Technology in Satellite Navigation (Beijing Institute of Technology), Ministry of Education, Beijing 100081, China

**Keywords:** interrupted sampling repeater jamming, digital radio frequency memory, radar waveform design, jamming perception, adaptive transmitting

## Abstract

The interrupted sampling repeater jamming (ISRJ) based on a digital radio frequency memory (DRFM) device is a new type of coherent jamming. This kind of jamming usually occurs as main-lobe jamming and has the advantages of low power requirements and easy parameter adjustment, posing a serious threat to the modern radar systems. In order to suppress the ISRJ, this paper proposes an adaptive transmitting scheme based on a phase-coded signal. The scheme firstly performs jamming perception to estimate the jamming parameters, then, on this basis, optimizes the waveform with genetic algorithm. With the optimized waveform, the jamming signal is orthogonal to the target echo, thus it can be easily suppressed with pulse compression. Simulation experiments are performed to verify the effectiveness of the scheme and the results suggest that the peak-to-side-lobe ratio (PSR) and integrated side-lobe level (ISL) of the pulse compression can be improved by about 16 dB and 15 dB, respectively, for the case where the jamming-to-signal ratio (JSR) is 13 dB.

## 1. Introduction

The application of digital radio frequency memory (DRFM) devices has greatly improved the performance and efficiency of radar electronic countermeasures (ECM) systems [1,2,3]. Based on the abilities of intercepting and storing radar transmitting signals, many new jamming strategies have been proposed [4,5,6,7]. Among them, interrupted sampling repeater jamming (ISRJ) has attracted extensive attention [8,9,10,11,12,13,14]. There are two main intercepting modes for DRFM devices, i.e., full-pulse sampling mode and interrupted sampling mode. Based on the latter, an ISRJ jammer intercepts parts of the radar signal and retransmits them for several times at a current pulse repeat interval (PRI). Therefore, the jamming signal is coherent with the radar transmission and partial processing gain can be obtained from signal processing such as pulse compression and coherent integration, greatly reducing the requirements on transmitting power. Thus the jammer can be more easily installed in small platforms such as unmanned aerial vehicles (UAVs) to form a main-lobe jamming, which increases the difficulty of radar antijamming [8]. Meanwhile, because of the digital processing capability of a DRFM system, the jammer can easily adjust the jamming parameters (such as the intercepting positions and intercepting durations, etc.) to change the distribution of the grating lobes of pulse compression, by which different jamming effects can be achieved.

ISRJ was first proposed by [9,10] in 2006, respectively (it was named as Chopping & Interleaving (C&I) jamming in [9]). According to these references, the jammer intercepts a slice of the radar transmission and retransmits it multiple times. This process will be conducted for several cycles until the falling edge of the signal is detected. After pulse compression, the jamming will appear as multiple false target groups and each of them consists of a main false target and several symmetrically distributed secondary false targets. By changing the intercepting duration and number of retransmitting times, the jamming can achieve effects of both deception and suppression. A modified strategy named interrupted sampling direct jamming (ISDJ) was also proposed in [10]. For this kind of jamming, the intercepted slices were retransmitted for only one time, whereas more slices can be intercepted. Therefore, the jamming will have only one false target group (after pulse compression), which consists of a stronger main false target and more secondary false targets. Hereafter, more publications were involved in this topic and most of them focused on jamming performance analysis and jamming strategy optimization [11,12,13]. For instance, Wang et al discussed the mathematical principle of ISRJ [11]; Li et al derived the connection between the intercepting duration and the jamming power [12] and studied the operating distance of the jammer based on coherent jamming principle [13].

In contrast, the electronic counter-countermeasures (ECCM) methods for ISRJ have not been fully studied. Some modern signal processing methods were attempted to filter out the jamming. Among them, the time-frequency (TF) analysis has been widely used. Especially, an ISRJ suppression method for dechirping radar was proposed in [15]. By making use of the discontinuity of ISRJ in the time-frequency domain, a band-pass filter was designed to suppress the jamming. However, this method requires a clear separation between adjacent false targets of the ISRJ. While in [16], the jamming was first reconstructed with the estimated jamming parameters, and then an adaptive CLEAN algorithm was employed to cancel the jamming. Despite their effectiveness, almost all of these methods are passive schemes, which means, countermeasures are taken after jamming enter the receiver. However, in the changing battlefield environment, these passive schemes require quite a lot of system resources while achieve limited performances.

For the above considerations, modern radar systems should have the ability of jamming perception [17,18,19], thus be able to choose the optimal antijamming method. Hence in this paper, an adaptive transmitting scheme based on a phase-coded signal was proposed. The scheme first carries out jamming perception to estimates the ISRJ parameters of jammer intercepting positions and intercepting durations, then performs waveform optimization with the estimated jamming information. With the optimized waveform, the intercepted jamming signal is orthogonal to the target echo, hence can be suppressed with pulse compression. Based on the iteration of jamming perception and waveform design, a dynamic active ISRJ suppression scheme can be formed. The structure of the paper is as follows: in Section 2, the mechanism of ISRJ is introduced and the operators of ISRJ are deduced. On this basis, the optimal antijamming waveform is given based on eigenvalue decomposition. In Section 3, the adaptive transmitting scheme for phase-coded signal is introduced, and the two key steps of jamming perception and waveform optimization are described in detail. In Section 4, simulation experiments are carried out to quantitatively evaluate the jamming suppression performances. Finally in Section 5, the main conclusions of the paper are summarized. The main notations used in this paper are defined in the following table (Table 1).

## 2. The Mechanism of ISRJ

### 2.1. The Operator Representation of Jamming Signal

**Figure 1:**
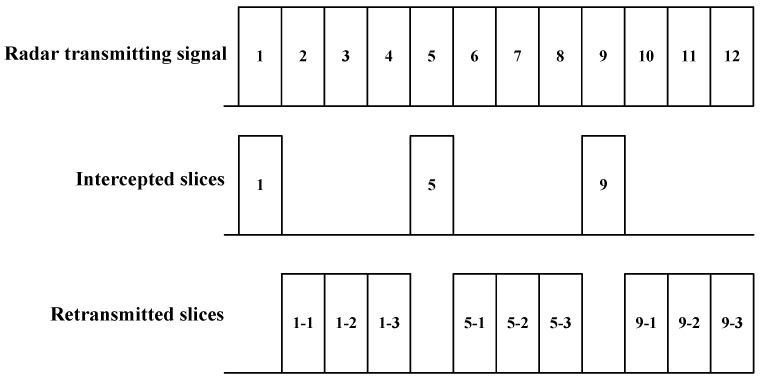
Mechanism of interrupted sampling repeater jamming (ISRJ).

The mechanism of ISRJ is shown in Figure 1. After detecting the rising edge of the radar transmitting signal, the jammer will intercept a slice based on the set strategy, and then delays and retransmits the slice for multiple times. The process of intercepting and retransmitting is repeated for several times until the falling edge of the signal is detected. Therefore, the ISRJ is actually emitting a partial sampled and delayed version of the radar transmitted signal.

Assuming the transmitted signal vector is $s={\left[\begin{array}{cccc} s_{1} & s_{2} & \cdots & s_{n} \end{array}\right]}^{T}$, where the subscript ‘*n*’ is the number of signal samples. Then the intercepted signal $x_{I}$ can be expressed as:

(1) $$\begin{array}{c} x_{I}=As\\ i.e.,\\ \left[\begin{array}{c} x_{1}\\ x_{2}\\ \vdots \\ x_{n} \end{array}\right]=\left[\begin{array}{cccc} a_{11} & 0 & \cdots & 0\\ 0 & a_{22} & \cdots & 0\\ \vdots & \vdots & & \vdots \\ 0 & 0 & \cdots & a_{nn} \end{array}\right]\left[\begin{array}{c} s_{1}\\ s_{2}\\ \vdots \\ s_{n} \end{array}\right],\;a_{ii}\in \left\{0,1\right\}. \end{array}$$

where the sampling matrix A is a diagonal matrix and the values of the diagonal elements are ‘one’ or ‘zero’. The element ‘one’ corresponds to the intercepting period, while the element ‘zero’ corresponds to the retransmitting period. The specific structure of A (i.e., the pattern of zeros and ones) is related to the ISRJ strategy.

Compared to the radar transmitting signal, the retransmitted signal has a time delay. By making use of the characteristics of Fourier transform, namely:

(2) $$\mathrm{fft}\left[s\left(t-\tau_{0}\right)\right]=\mathrm{fft}\left[s\left(t\right)\right]e^{-j2\pi f\tau_{0}}.$$

where fft[⋅] means fast Fourier transform (FFT) and $\tau_{0}$ is the time delay.

The retransmitting of the intercepted signal can be described by the process of ‘FFT-phase shift-IFFT (inverse fast Fourier transform)’, and each of the steps can be expressed as:

- FFT to the intercepted signal:
  (3) $$\begin{array}{c} X=Fx_{I}\\ i.e.,\\ \left[\begin{array}{c} X_{1}\\ X_{2}\\ \vdots \\ X_{n} \end{array}\right]=\left[\begin{array}{cccc} e^{-j2\pi f_{1}t_{1}} & e^{-j2\pi f_{1}t_{2}} & \cdots & e^{-j2\pi f_{1}t_{n}}\\ e^{-j2\pi f_{2}t_{1}} & e^{-j2\pi f_{2}t_{2}} & \cdots & e^{-j2\pi f_{2}t_{n}}\\ \vdots & \vdots & & \vdots \\ e^{-j2\pi f_{n}t_{1}} & e^{-j2\pi f_{n}t_{2}} & \cdots & e^{-j2\pi f_{n}t_{n}} \end{array}\right]\left[\begin{array}{c} x_{1}\\ x_{2}\\ \vdots \\ x_{n} \end{array}\right]. \end{array}$$
  where F is the Fourier transform coefficient matrix and X is the spectrum of the intercepted signal.
- Multiply X by the phase factor (take ‘one retransmitting’ as an example):
  (4) $$\begin{array}{c} Y=TX\\ i.e.,\\ \left[\begin{array}{c} Y_{1}\\ Y_{2}\\ \vdots \\ Y_{n} \end{array}\right]=\left[\begin{array}{cccc} e^{-j2\pi f_{1}\tau } & 0 & \cdots & 0\\ 0 & e^{-j2\pi f_{2}\tau } & \cdots & 0\\ \vdots & \vdots & & \vdots \\ 0 & 0 & \cdots & e^{-j2\pi f_{n}\tau } \end{array}\right]\left[\begin{array}{c} X_{1}\\ X_{2}\\ \vdots \\ X_{n} \end{array}\right]. \end{array}$$
  where T is the phase matrix corresponding to retransmitting delay τ and Y is the phase shifted spectrum.
- IFFT to the phase shifted spectrum:
  (5) $$\begin{array}{c} J=F_{I}Y\\ i.e.,\\ \left[\begin{array}{c} J_{1}\\ J_{2}\\ \vdots \\ J_{n} \end{array}\right]=\left[\begin{array}{cccc} e^{j2\pi f_{1}t_{1}} & e^{j2\pi f_{2}t_{1}} & \cdots & e^{j2\pi f_{n}t_{1}}\\ e^{j2\pi f_{1}t_{2}} & e^{j2\pi f_{2}t_{2}} & \cdots & e^{j2\pi f_{n}t_{2}}\\ \vdots & \vdots & & \vdots \\ e^{j2\pi f_{1}t_{n}} & e^{j2\pi f_{2}t_{n}} & \cdots & e^{j2\pi f_{n}t_{n}} \end{array}\right]\left[\begin{array}{c} Y_{1}\\ Y_{2}\\ \vdots \\ Y_{n} \end{array}\right]. \end{array}$$
  where $F_{I}$ is the inverse Fourier transform coefficient matrix and J is the retransmitted jamming signal.

In summary, the jamming signal can be expressed as:

(6) $$J=F_{I}TFAs.$$

where $\Gamma =F_{I}TFA$ is defined as the jamming operator. When considering the case of a slice is retransmitted for multiple times, the jamming operator can be expressed as:

(7) $$\Gamma =F_{I}TFA+F_{I}T^{2}FA\cdots +F_{I}T^{M}FA.$$

where M is the number of retransmitting times of each jamming slice.

### 2.2. The Optimal Waveform for ISRJ Suppression

According to previous analysis, the jamming signal is the output of jamming operator on radar transmitting signal. The process can be expressed as:

(8) J=Γs.

According to the matrix theory, we may have:

(9) Γu=λu.

where u is the eigenvector of Γ and λ is the corresponding eigenvalue.

Therefore, in theory, the jamming signal can be suppressed by taking the eigenvector associated with some zero eigenvalue (or the linear combination of eigenvectors associated with multiple zero eigenvalues) as the transmitting signal.

To analyze the characteristics of the eigenvectors associated with zero eigenvalues, we still take‘one retransmitting’ as an example. Then the jamming operator can be rewritten as follows according to Equation (6):

(10) $$\begin{array}{cc} \Gamma & =F_{I}TFA\\ & =EA\\ & =\left[\begin{array}{llll} e_{11} & e_{12} & \cdots & e_{1N}\\ e_{21} & e_{22} & \cdots & e_{2N}\\ \vdots & \vdots & \ddots & \vdots \\ e_{N1} & e_{N2} & \cdots & e_{NN} \end{array}\right]A. \end{array}$$

where $E=F_{I}TF$, $e_{ij}=\sum_{k=1}^{N}e^{j2\pi f_{k}\left[\left(i-j\right)\Delta t-\tau \right]}$, Δt is the time sampling interval, τ is the retransmitting delay and N is the total number of the signal samples.

Assuming that the jammer intercepts L signal samples at a time, then the sampling matrix can be expressed as:

(11) $$A=\left[\begin{array}{cccccccccc} 1 & & & & & & & & & \\ & \overset{\left(L\right)}{\ddots } & & & & & & & & \\ & & 1 & & & & & & & \\ & & & 0 & & & & & & \\ & & & & \overset{\left(L\right)}{\ddots } & & & & & \\ & & & & & 0 & & & & \\ & & & & & & 1 & & & \\ & & & & & & & \overset{\left(L\right)}{\ddots } & & \\ & & & & & & & & 1 & \\ & & & & & & & & & \ddots \end{array}\right].$$

Thus the operator matrix Γ can be expressed as:

(12) $$\Gamma =\left[\begin{array}{cccccccccc} e_{11} & \overset{\left(L\right)}{\cdots } & e_{1L} & 0 & \overset{\left(L\right)}{\cdots } & 0 & e_{1\left(2L+1\right)} & \overset{\left(L\right)}{\cdots } & e_{1\left(3L\right)} & \cdots \\ e_{21} & \cdots & e_{2L} & 0 & \cdots & 0 & e_{2\left(2L+1\right)} & \cdots & e_{2\left(3L\right)} & \cdots \\ \vdots & & \vdots & \vdots & & \vdots & \vdots & & \vdots & \vdots \\ e_{N1} & \cdots & e_{NL} & 0 & \cdots & 0 & e_{N\left(2L+1\right)} & \cdots & e_{N\left(3L\right)} & \cdots \end{array}\right].$$

Let λ be the eigenvalues of Γ, then the following equation can be obtained [20]:

(13) $$\det\left(\Gamma -\lambda I\right)={\left(-\lambda \right)}^{\alpha }{\left(\sum_{i=1}^{N}e^{-j2\pi f_{i}\tau }\right)}^{N-\alpha }=0.$$

where α>1 is the total number of the intercepted samples by the jammer.

Equation (13) shows that the operator matrix of the jamming have zero eigenvalues. Therefore, we will analyze the characteristics of eigenvectors associated with zero eigenvalues.

Firstly, since both F and $F_{I}$ are reversible, they are full rank. Secondly, it is easy to test that the phase matrix T is full rank as well. Therefore, their product, that is, the matrix E is full rank, which means the column vectors of E are linearly independent. Then constitutes a new matrix B with the nonzero column vectors in Γ, and the new matrix is also full rank.

Therefore, solving the matrix equation:

(14) Bx=0.

we have the solution x=0.

The optimal waveform we want should meet:

(15) Γx=0.

Substituting Equation (12) into Equation (15), we have the following equation group:

(16) $$\{\begin{array}{c} e_{11}x_{1}\cdots +e_{1L}x_{L}+0\cdot x_{L+1}\cdots +0\cdot x_{2L}\cdots +e_{1N}x_{N}=0\\ e_{21}x_{1}\cdots +e_{2L}x_{L}+0\cdot x_{L+1}\cdots +0\cdot x_{2L}\cdots +e_{2N}x_{N}=0\\ \vdots \\ e_{N1}x_{1}\cdots +e_{NL}x_{L}+0\cdot x_{L+1}\cdots +0\cdot x_{2L}\cdots +e_{NN}x_{N}=0 \end{array}.$$

It could be seen from Equation (16) that:

1. For thevariables whose coefficient are not zero, the equation holds only if the variables satisfy $x_{i}=0$.
2. While for the variables whose coefficient are zero, the equation holds for any value of variables $x_{i}$.

According to the above analysis, the optimal waveform should be zero during the intercepting durations of the jammer; in other words, the optimal waveform is time discontinuous. Therefore, for convenience, we call it the time-discontinuous waveform (TDW).

For the case of multiple retransmitting, the difference lies only in the structure of the sampling matrix A:

(17) $$A=\left[\begin{array}{cccccccccc} 1 & & & & & & & & & \\ & \overset{\left(L\right)}{\ddots } & & & & & & & & \\ & & 1 & & & & & & & \\ & & & 0 & & & & & & \\ & & & & \overset{\left(ML\right)}{\ddots } & & & & & \\ & & & & & 0 & & & & \\ & & & & & & 1 & & & \\ & & & & & & & \overset{\left(L\right)}{\ddots } & & \\ & & & & & & & & 1 & \\ & & & & & & & & & \ddots \end{array}\right].$$

Which means the jammer intercepts L signal samples at a time and retransmits them for M times.

For this matrix, the above analysis also applies, hence the same conclusion can be drawn, i.e., the optimal radar waveform should transmit when the jammer is not sampling.

## 3. Adaptive Transmitting Scheme for ISRJ Suppression

**Figure 2:**
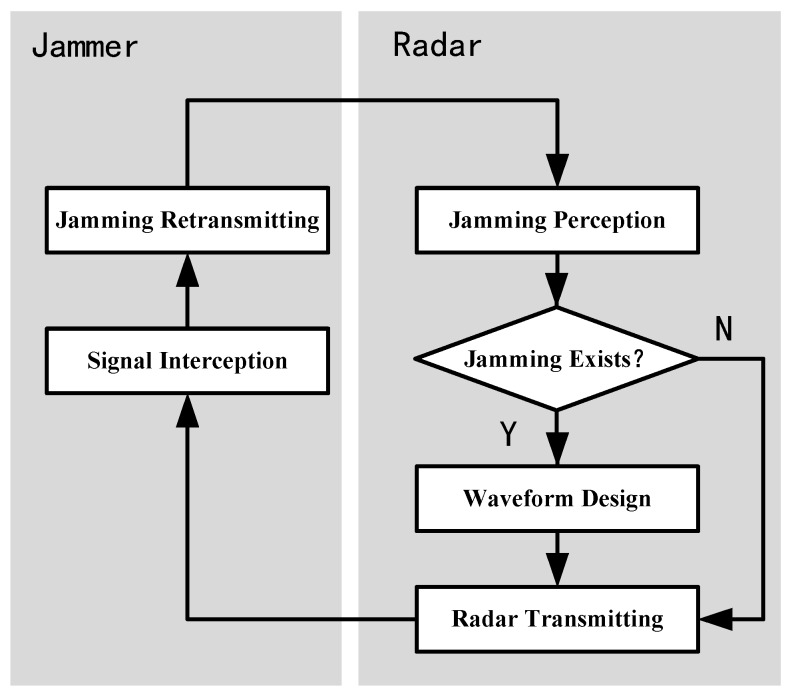
Flow chart of adaptive transmitting scheme.

In this section, an adaptive transmitting scheme for ISRJ suppression is presented. The flowchart of the scheme is shown in Figure 2. The scheme adaptively adjusts the transmitting waveform according to the dynamic jamming environment, thus realizing the dynamic game between the radar and the jammer.

The detailed interpretations are as follows:

1. At the beginning, the radar transmits a predesigned waveform according to the mission;
2. When the ISRJ jammer starts to work, it intercepts and retransmits the radar signal according to the set strategy;
3. The radar carries out jamming perception to determine whether the jamming exists:
  - if there is no jamming, the radar maintains the transmitting waveform unchanged;
  - if there is jamming in the echo, the jamming parameters estimation is performed;
4. The radar optimizes the waveform with the jamming parameters and transmits the optimized waveform in the next PRI.

**Figure 3:**
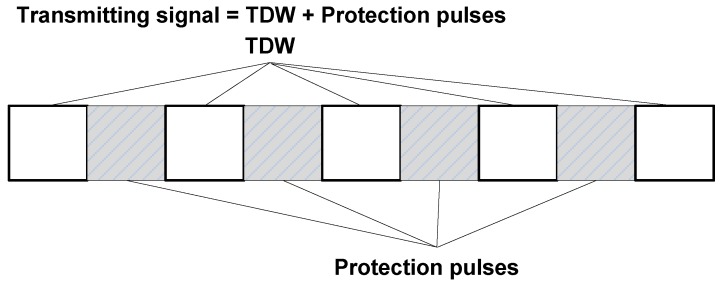
Structure of transmitting waveform for ISRJ suppression.

According to the analysis of previous section, it is theoretically possible to achieve ISRJ suppression by transmitting TDW whose discontinuous positions are consistent with the jammer intercepting periods. However, a TDW may imply complex hardware and large radar operational bandwidth. Besides, the jammer will always detect the rising edge of the signal, so the simple TDW will cause the jammer to change the strategy, resulting in a failure of the jamming suppression ability of the designed waveform. Therefore, we shall insert some protection pulses at the discontinuous intervals of a TDW, making the whole waveform continuous in time. For the purposes of jamming suppression, the protection pulses should be orthogonal to the TDW. With this waveform, the jammer will sample and retransmit the protection pulses, thus the jamming signal can be easily suppressed with pulse compression. The structure of the transmitting waveform is shown in Figure 3.

### 3.1. JammingPerception

In order to effectively jam the radar, the energy of the jamming signal generally needs to be greater than the target echo. ISRJ can achieve some coherent processing gain, thereby reducing the requirement for transmitting power. But with respect to the target echo, the processing gain achievable is small. This means that the jamming-to-noise ratio (JNR) of ISRJ needs to be significantly larger than the signal-to-noise ratio (SNR) of the target echo. For example, if each jamming slice is retransmitted once (in which case the jamming signal can achieve greatest processing gain), the peak amplitude of the jamming signal after pulse compression will be the same as that of the target when the JNR is two times of the SNR. To achieve better jamming effect, the JNR of ISRJ generally requires to be higher, which provides favorable condition for jamming perception.

The purpose of jamming perception is to estimate the intercepting positions and durations of an ISRJ jammer, and provide the necessary prior knowledge for the antijamming waveform design. This is essentially a problem of target detection and parameter estimation in noise. Similar problems have been extensively studied in the fields of target detection [21,22], edge detection and time of arrival (TOA) estimation [23]. While in this paper, the purpose is achieved with steps of wavelet denoising, amplitude difference and peak detection. These methods has been widely used in different areas [24,25,26,27], hence will not be described in detail here. According to the subsequent simulations, the jamming parameters can be accurately estimated when JNR is greater than 0dB.Besides, the JNR can be further increased by pulse accumulation if the conditions permit.

### 3.2. Joint Design of TDW and Protection Pulse

A phase-coded signal has the advantages of simple implementation and good clutter suppression performance [28,29], therefore is widely used in radar transmission. In this section, the binary phase-coded signal is taken as an example to illustrate the joint design of TDW and protection pulse.

Assume that the discontinuous positions of TDW are defined by:

(18) $$\Omega =\overset{K}{\underset{k=1}{\cup }}\left(t_{k1},t_{k2}\right).$$

where *K* is the number of discontinuous positions; $t_{k1}$ and $t_{k2}$ are the start time and end time of the *k*th discontinuous position.

Then the TDW can be expressed as:

(19) $$x=As_{1}.$$

where $s_{1}$ is a binary phase-coded sequence; x is the TDW; A is the sampling matrix, whose columns corresponding to the time defined in Ω are zero and the other elements are one.

Similarly, the protection pulse can be expressed as:

(20) $$p=C_{A}s_{2}.$$

where $s_{2}$ is another binary phase-coded sequence; p is the protection pulse and $C_{A}$ is the sampling matrix of protection pulse which is complementary to A, i.e., the columns corresponding to the time defined in Ω are one and the other elements are zero.

In waveform design research, the peak to side-lobe ratio (PSR) and integrated side-lobe level (ISL) of the autocorrelation function are usually used to evaluate the pulse compression performance of a waveform, while those of the cross-correlation function are usually used to evaluate the orthogonality of a pair of waveforms [30,31]. The two functions can be expressed as:

The autocorrelation function of x:

(21) $$\gamma_{xx}(m)=\sum_{k=0}^{N-1}x(k)x*\left(k+m\right).$$

The cross-correlation function of ***x*** and ***p***:

(22) $$\gamma_{xp}(m)=\sum_{k=0}^{N-1}x(k)p*\left(k+m\right).$$

However, for the situation considered in this paper, both the target echo and jamming signal are present in the received signal. Therefore, the autocorrelation characteristic of TDW alone cannot fully reflect the PSR and ISL of the signal after pulse compression. Considering that the jamming signal is essentially the retransmission of the protection pulse, therefore, it is necessary to consider the PSR and ISL characteristics of the summation of the autocorrelation and the cross-correlation functions. That is, the PSR of the sum function should be as large as possible while the ISL should be as small as possible.

The summation of the autocorrelation and the cross-correlation functions is:

(23) $$\chi \left(m\right)=\gamma_{xx}\left(m\right)+\gamma_{xp}\left(m\right).$$

Then the PSR and ISL can be defined as:

(24) $$PSR=\frac{{\left|\chi \left(0\right)\right|}^{2}}{\underset{\left|k\right|>\delta }{\max}{\left|\chi \left(k\right)\right|}^{2}}.$$

(25) $$ISL=\frac{\sum_{\left|k\right|>\delta }{\left|\chi \left(k\right)\right|}^{2}}{\sum_{\left|k\right|\leq \delta }{\left|\chi \left(k\right)\right|}^{2}}.$$

where δ is the boundary of the main lobe of the autocorrelation function.

The cost function can then be expressed as the weighted sum of the two indicators:

(26) $$F=\frac{\alpha }{PSR}+\left(1-\alpha \right)ISL.$$

where α is an adjusting factor.

Many modern optimization methods (such as water-filling algorithm, simulated annealing method, particle swarm optimization, compressed sensing etc.) have been used to solve such problems [32,33,34,35,36]. In this paper, the genetic algorithm (GA) [36] is used to optimize the waveform.

## 4. Simulations

In order to verify the effectiveness of the proposed scheme, simulation experiments are performed with the parameters shown in Table 2.

The system uses an m-sequence-based binary phase-coded signal as the transmitting signal. The code number is 100 and the code width is 0.1 μs. The jammer switches to ‘intercepting and retransmitting’ mode after detecting the rising edge of the radar signal. The intercepting duration is set 1 μs and each of the intercepted slice is retransmitted for twice. Therefore, the jammer can intercept 4 jamming slices in the whole pulse duration.

**Figure 4:**
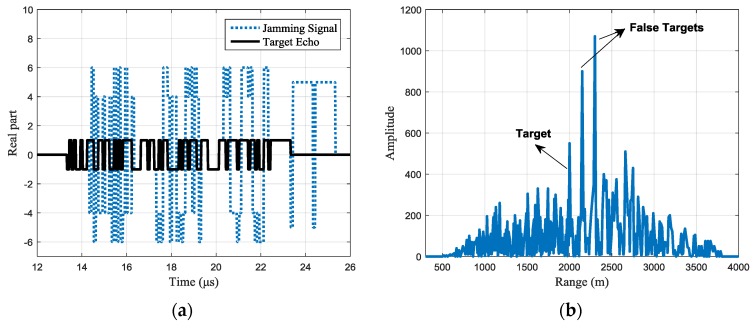
Jamming effect of common binary phase-coded signal. (**a**) The real part of the received signals and (**b**) pulse compression of the received signals.

Assuming the jamming-to-signal ratio (JSR) is 15 dB, then the real part and pulse compression result of the received signals are shown in Figure 4. In Figure 4a, the black line shows the real part of the target echo, while the blue line shows the real part of the jamming signal.

The pulse compression result is shown in Figure 4b. With the set simulation parameters, the jamming signal forms two false targets after pulse compression. Since the jamming signal is just the retransmission of parts of the whole signal, the available processing gain is smaller than that of the target echo’s. Hence, the peak JSR is reduced by about 7 dB after pulse compression, but the false targets are still strong enough to cause false alarms. Besides, the random peaks caused by the superposition of the side lobes will seriously affect the detection of the real target, either.

**Figure 5:**
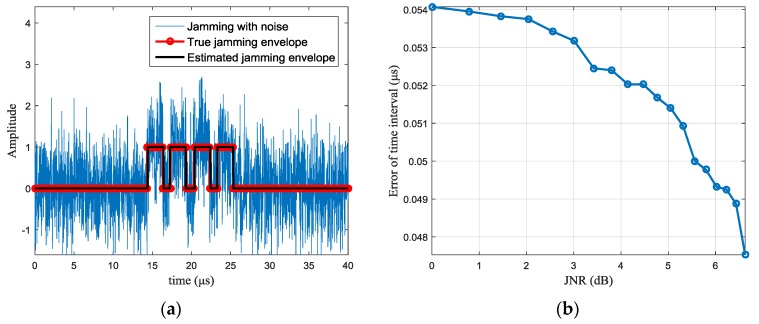
Simulations of jamming perception (**a**) estimation results of jamming parameters with jamming-to-noise ratio (JNR) =0 dB (**b**) jamming parameters estimation errors VS. JNR.

The simulations of jamming perception are shown in Figure 5. With wavelet denoising, amplitude difference and peak detection, the typical results of jamming parameters estimation are shown in Figure 5a, in which the JNR is set 0 dB and the red curve represents the true jamming envelope, while the black curve is the estimated jamming envelope. The discontinuous position between each segment of the jamming signal corresponds to the intercepting period of the jammer. With different JNR, the estimation error of the intercepting duration is shown in Figure 5b. It can be seen that, when the JNR is 0 dB, the estimation error is about 0.054 μs (the value is the average of 100 Monte Carlo simulations), which is equivalent to 2 or 3 sampling points according to the sampling rate. The error is acceptable for subsequent waveform design.

**Figure 6:**
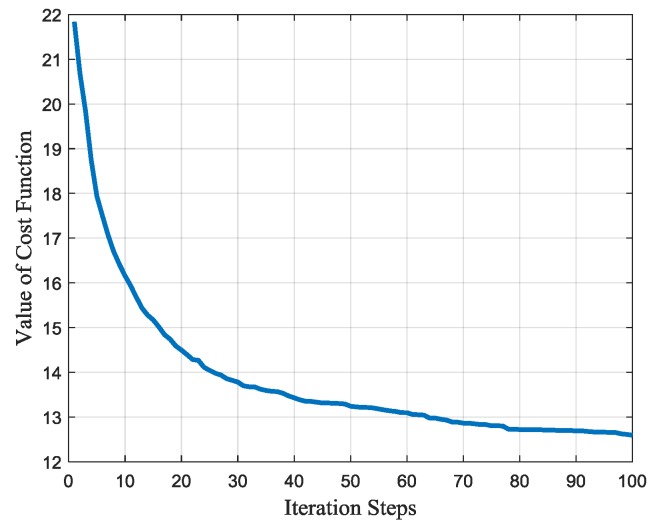
The convergence process of the waveform design algorithm.

With the estimated jamming parameters, the cost function can be given with Equations (21)–(26) and solved with genetic algorithm (GA). Performing 100 Monte Carlo simulations, the average value of the cost function in each iteration step is shown in Figure 6. The iteration is stopped when the generation number reaches 100 and the cost function is convergent to the value of about 13.

**Figure 7:**
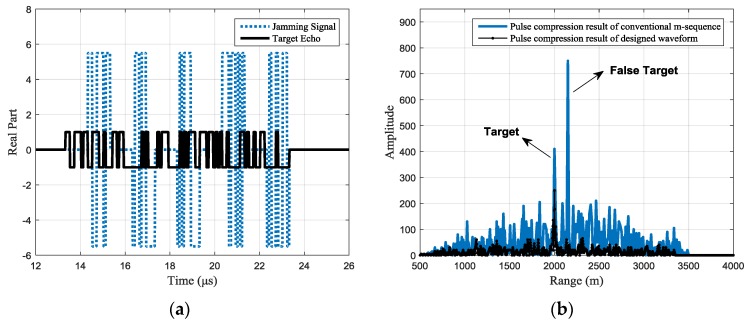
Jamming suppression result of a typical designed waveform (the intercepting duration is 1 μs and each jamming slice is retransmitted once): (**a**) the real part of the received signals with a typical designed waveform (**b**) pulse compression result of the received signal.

**Figure 8:**
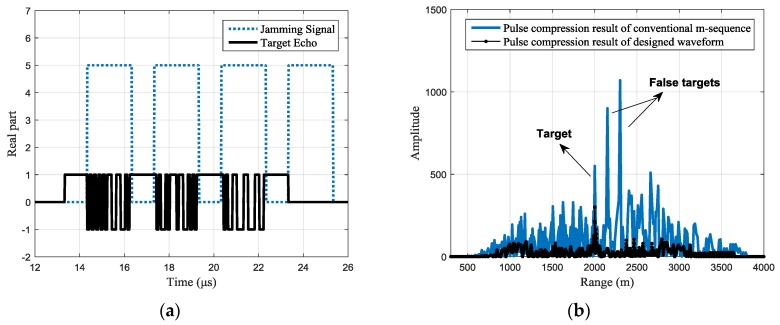
Jamming suppression result of a typical designed waveform (the intercepting duration is 1 μs and each jamming slice is retransmitted twice): (**a**) the real part of the received signals with a typical designed waveform (**b**) pulse compression result of the received signal.

**Figure 9:**
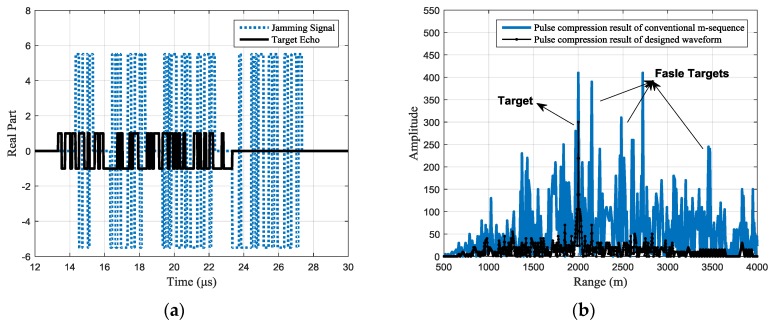
Jamming suppression result of a typical designed waveform (each jamming slice is retransmitted for different times): (**a**) the real part of the received signals with a typical designed waveform (**b**) pulse compression result of the received signal.

For different jamming scenarios, the typical designed waveforms can be seen from the target echo shown in Figure 7a, Figure 8a, and Figure 9a with the black line. Under the same jams shown with the blue dotted line. In Figure 7, each jamming slice is retransmitted once; in Figure 8, each jamming slice is retransmitted twice, while in Figure 9, the jamming slices are retransmitted for different times, where the first slice is retransmitted once, the second slice is retransmitted twice, and so on. It should be noted that the figure only gives a typical result of the joint design process. Signals with different codes may be outputted in the 100 simulations.

The pulse compression results of the received signal are shown in Figure 7b, Figure 8b and Figure 9b, where the black line shows the pulse compression result of the received signal with the designed waveform. While in the same figure, the pulse compression result of received signal with the conventional phase-coded signal is shown with the blue line for comparison. It can be seen that, compared with the conventional m-sequence based binary phase-coded waveform, the false targets are effectively suppressed with the designed waveform and the side lobes are also significantly reduced.

However, because the protection pulse in the designed waveform cannot provide any processing gain for the target echo, there is a target energy loss relative to the conventional waveform. The loss is related to the jamming strategy. When each intercepted slice is retransmitted for only one time, the length of the total intercepted signal can be half of the transmitting pulse duration. At this point, the target energy loss will reach the maximum, i.e., 3 dB.

**Figure 10:**
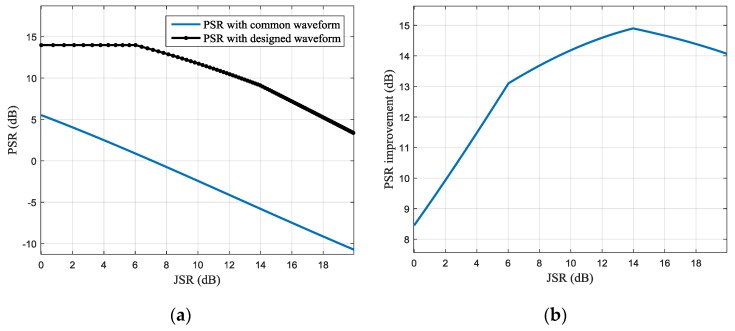
Peak-to-side-lobe ratio (PSR) of the pulse compression result under different jamming-to-signal ratio (JSR) (**a**) PSR of the pulse compression result with common and designed waveforms (**b**) PSR improvement with different JSR.

**Figure 11:**
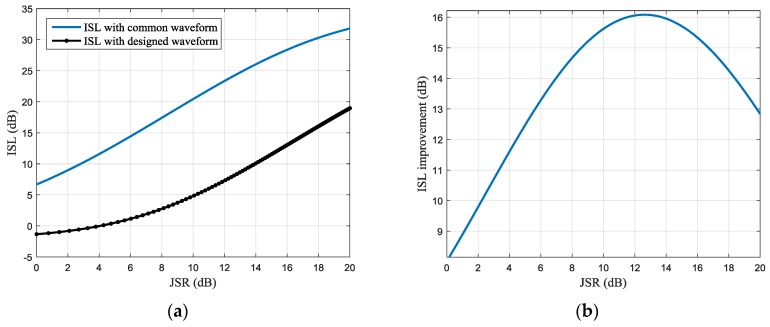
Integrated side-lobe level (ISL) of the pulse compression result under different JSR (**a**) ISL of the pulse compression result with common and designed waveforms (**b**) ISL improvement with different JSR.

The average jamming suppression performance of the obtained 100 waveforms with different JNR conditions is shown in Figure 10 and Figure 11. In Figure 10a, the blue and black lines, respectively, present the PSR of the pulse compression results with the conventional and designed waveforms, and the PSR improvement with different JSR is shown in Figure 10b. It can be seen that with the increase of the JSR, the JSR improvement will increase first and then decrease. For the simulation parameters of this paper, the optimal PSR improvement is about 15 dB, which can be obtained when the JSR is about 14 dB.

Similarly, the ISL of the pulse compression result is shown in Figure 11 and the optimal ISL improvement is about 16 dB, which is obtained at the JSR of 12.8 dB for the same simulation parameters.

The results suggest that the designed waveform has an obvious ISRJ suppression effect.

## 5. Conclusions

In order to suppress the ISRJ injected from the main lobe of the radar antenna, an adaptive transmitting scheme is proposed in this paper. Based on an ordinary single-channel radar, the scheme first estimates the intercepting positions and durations of the ISRJ with jamming perception technologies, then performs waveform optimization for ISRJ suppression (the designed waveform is named TDW). By inserting some protection pulses orthogonal to the TDW at the jammer intercepting positions, the processing gain obtained by the jamming signal can be greatly reduced, thereby suppressing the jamming performance. The simulation results show that the jamming parameters can be effectively estimated when the JNR is greater than 0dB. The designed antijamming waveform can achieve a JSR improvement of more than 10 dB when the input JSR is less than 20 dB, while the target energy loss less than 3 dB.

## Figures and Tables

**Table 1:** Main notations used in the paper.

Symbols	Interpretation
s	Transmitted signal vector
$x_{I}$	Intercepted signal vector
A	Jammer sampling matrix
$\tau_{0}$	Time delay
F	Fourier transform coefficient matrix
X	Spectrum of the intercepted signal
τ	Retransmitting delay
T	Phase matrix corresponding to the retransmitting delay
Y	Phase shifted spectrum
$F_{I}$	Inverse Fourier transform coefficient matrix
J	Jamming signal vector
Γ	Jamming operator
M	Retransmitting time of each jamming slice
u	Eigenvector of Γ
λ	Eigenvalueof Γ
$\Omega =\overset{K}{\underset{k=1}{\cup }}\left(t_{k1},t_{k2}\right)$	Discontinuous positions set
K	Number of discontinuous positions
$t_{k1}$, $t_{k2}$	Start time and end time of the kth discontinuous position
$s_{1}$, $s_{2}$	Binary phase-coded sequence
x	Time-discontinuous waveform
p	Protection pulse vector
$C_{A}$	Sampling matrix of protection pulse, complementary to A
F	Cost function for waveform optimization

**Table 2:** Simulation parameters for ISRJ.

Parameter	Value
Transmitting signal	Binary phase-coded (m-sequence)
Carrier frequency	6 GHz
Code width	0.1 μs
Code number	100
Sampling rate	50 MHz
Sampling points	2048
Intercepting duration	1 μs
Retransmitting time	2
